# Biosynthetic Diversity of Marine-Derived *Streptomyces* Natural Products: Integrating Genome Mining, Multi-Omics, and Translational Drug Discovery Strategies

**DOI:** 10.3390/md24090324

**Published:** 2026-09-17

**Authors:** Monthon Lertcanawanichakul, Phuangthip Bhoopong, Tuanhawanti Sahabuddeen, Patchara Pedpradab, Husna Madoromae, Sueptrakool Wisessombat, Attarat Pattanawongsa, Nuttapon Songnaka

**Affiliations:** 1 School of Allied Health Sciences, Walailak University, Nakhon Si Thammarat 80160, Thailand; bphuangt@wu.ac.th (P.B.); tuanhawanti@gmail.com (T.S.); husna.md@mail.wu.ac.th (H.M.); sueptrakool.wi@wu.ac.th (S.W.); 2 Food Technology and Innovation Center of Excellence, Walailak University, Nakhon Si Thammarat 80160, Thailand; 3 Faculty of Science and Technology, Rajamangala University of Technology Trang, Trang 92150, Thailand; ppedpradab@gmail.com; 4 School of Pharmacy, Walailak University, Nakhon Si Thammarat 80160, Thailand; pattarat@wu.ac.th (A.P.); nuttapon.so@wu.ac.th (N.S.)

**Keywords:** marine *Streptomyces*, biosynthetic gene clusters, genome mining, natural products, polyketides, non-ribosomal peptides, synthetic biology, metabolomics, translational drug discovery

## Abstract

Marine-derived *Streptomyces* are among the most prolific producers of structurally diverse and biologically active natural products. Adaptation to unique marine environments, including deep-sea sediments, hydrothermal vents, mangrove ecosystems, marine invertebrates, and hypersaline habitats, has promoted the evolution of specialized biosynthetic systems capable of generating a broad spectrum of secondary metabolites. These metabolites include polyketides, non-ribosomal peptides (NRPs), ribosomally synthesized and post-translationally modified peptides (RiPPs), terpenoids, alkaloids, and hybrid compounds with significant antibacterial, antifungal, antiviral, antiparasitic, anti-inflammatory, and anticancer activities. Recent advances in genome sequencing, bioinformatics, genome mining, metabolomics, synthetic biology, and artificial intelligence (AI)-assisted discovery have substantially expanded marine natural product research by enabling the identification and prioritization of previously inaccessible biosynthetic gene clusters (BGCs). However, major challenges remain, including silent biosynthetic pathways, low cultivation efficiency, rediscovery of known compounds, metabolite yield instability, dereplication bottlenecks, and limited ecological interpretation. These constraints continue to impede the translation of biosynthetic potential into pharmaceutical applications. This review summarizes current strategies for marine natural product discovery and highlights emerging translational approaches integrating multi-omics technologies, pathway engineering, and AI-guided prioritization. Collectively, these advances provide a roadmap for advancing marine *Streptomyces* research from descriptive omics-based exploration toward experimentally validated and clinically relevant drug discovery.

## 1. Introduction

Marine ecosystems represent one of the largest reservoirs of microbial diversity on Earth, harboring microorganisms with substantial capacity to produce structurally unique secondary metabolites [1,2]. Among these, marine-derived *Streptomyces* have attracted considerable scientific attention due to their historical and ongoing contributions to natural-product discovery. These Gram-positive filamentous bacteria typically possess large linear chromosomes enriched with biosynthetic gene clusters (BGCs) that encode a wide variety of natural products [3,4,5,6]. Terrestrial *Streptomyces* historically yielded many clinically important compounds, including streptomycin, chloramphenicol, tetracycline, and avermectin [5,6]; however, traditional terrestrial screening has faced diminishing returns due to rediscovery of known metabolites and limited chemical novelty [7,8].

Marine-derived *Streptomyces* have emerged as important sources of chemically diverse natural products, shaped by environmental conditions such as salinity, hydrostatic pressure, nutrient limitation, oxidative stress, and microbial competition [9,10,11]. These environmental conditions may contribute to the diversification of specialized biosynthetic pathways and adaptive metabolic systems, resulting in metabolites containing unusual halogenated moieties, sulfur groups, rare amino acids, macrocyclic structures, and highly modified polyketide scaffolds [10,12,13,14]. Many of these compounds exhibit potent antibacterial, antifungal, antiviral, antiparasitic, anti-inflammatory, and anticancer activities, underscoring their pharmaceutical potential [2,11,15,16,17].

Next-generation sequencing, genome mining, and advanced bioinformatics have revealed substantial biosynthetic potential in marine-derived *Streptomyces*, including numerous predicted biosynthetic gene clusters that are not readily expressed under standard laboratory conditions [12,13,14,15]. Tools such as antiSMASH, PRISM, BAGEL, DeepBGC, and BiG-SCAPE have demonstrated widespread cryptic or silent BGCs that remain inactive under standard laboratory conditions [13,14,18,19]. Strategies to activate these clusters—including co-cultivation, OSMAC approaches, ribosome engineering, CRISPR-Cas-mediated activation, epigenetic modulation, and heterologous expression—have become central to unlocking previously inaccessible metabolites [14,15,20,21].

Integration of metabolomics and molecular networking has accelerated dereplication and prioritized chemically novel compounds for further study [16,22]. Moreover, AI-assisted metabolite prediction and machine learning-based biosynthetic analyses are transforming natural product discovery into a data-driven discipline [17,23]. Despite these advances, several translational challenges remain: low cultivation efficiency, silent BGC expression, frequent rediscovery of known metabolites, unstable metabolite yields, and insufficient ecological and functional validation [14,15,24,25,26].

Synthetic biology approaches, including promoter engineering, pathway refactoring, and heterologous expression, allow scalable production and functional validation of bioactive molecules [20,21,27,28]. Additionally, integrative pipelines that combine genomics, metabolomics, and AI-guided prioritization are beginning to bridge the gap between discovery and application [23,29,30,31]. A critical synthesis integrating ecological adaptation, biosynthetic logic, genome-guided discovery, enzymatic diversification, and translational pharmaceutical perspectives in marine-derived Streptomyces remains limited but is important for evaluating their biosynthetic and translational potential [1,4,12,14,15,32,33,34].

Therefore, this review critically evaluates the biosynthetic diversity of marine-derived *Streptomyces* natural products and highlights emerging translational strategies that integrate genome mining, metabolomics, synthetic biology, and drug development. We emphasize the need for integrative and functionally validated approaches to unlock the full pharmaceutical potential of marine microbial biosynthetic systems [1,2,3,4,5,6,7,8,9,10,11,12,13,14,15,16,17,18,19,20,21,22,23,24,25,26,27,28,29,30,31,32,33,34,35,36,37,38,39,40,41,42,43,44,45,46,47,48,49,50].

## 2. Methodology

This review was conducted using a narrative-critical review approach focusing on the biosynthetic diversity, enzymatic mechanisms, genomic potential, metabolomics-guided discovery, and pharmaceutical applications of marine-derived *Streptomyces*. Relevant literature was identified through targeted searches of PubMed, Scopus, Web of Science, ScienceDirect, and Google Scholar databases. The literature search primarily covered publications from 1990 to 2026 using combinations of keywords including “marine *Streptomyces*”, “marine actinomycetes”, “biosynthetic gene clusters”, “marine natural products”, “genome mining”, “polyketides”, “non-ribosomal peptides”, “RiPPs”, “metabolomics”, “secondary metabolites”, “synthetic biology”, and “drug discovery”.

Only peer-reviewed articles published in English were included. Studies focusing exclusively on terrestrial actinomycetes without direct relevance to marine biosynthesis were excluded unless they provided essential comparative biosynthetic insights. Emphasis was placed on studies involving experimentally validated biosynthetic pathways, metabolite characterization, enzymatic tailoring reactions, genome-guided discovery strategies, synthetic biology approaches, and translational pharmaceutical applications.

The selected literature was critically analyzed to evaluate not only technological advances but also current limitations and conceptual gaps within the field. Particular attention was given to challenges involving silent biosynthetic gene clusters, metabolite rediscovery, annotation overinterpretation, low cultivation efficiency, dereplication bottlenecks, scalability limitations, and insufficient ecological interpretation. References were organized according to Vancouver style and aligned sequentially with their first citation in the text.

Unlike traditional narrative surveys focused predominantly on compound cataloging, this review emphasizes critical evaluation of current methodological limitations, translational barriers, and conceptual gaps within marine *Streptomyces* research. Particular attention is given to discrepancies between computational biosynthetic prediction and experimental validation, as well as the emerging need for integrative and functionally validated biosynthetic research frameworks. The search and selection process was designed to support a narrative-critical synthesis rather than a systematic review and therefore did not follow a formal systematic-review protocol or PRISMA framework.

## 3. Marine Streptomyces Diversity and Ecological Distribution

Marine-derived *Streptomyces* have been isolated from highly diverse ecological niches including marine sediments, deep-sea environments, hydrothermal vents, mangrove ecosystems, marine sponges, algae, seawater, and saline habitats [7,8,9]. These ecosystems represent chemically dynamic and highly competitive environments that appear to stimulate the evolution of specialized secondary metabolism. Marine sediments remain the most extensively investigated source of marine *Streptomyces*. Sediment-associated strains frequently produce structurally diverse polyketides, alkaloids, peptides, and hybrid metabolites exhibiting potent antibacterial and anticancer activities.

Deep-sea *Streptomyces* isolated from abyssal sediments and hydrothermal vents possess particularly distinctive biosynthetic capabilities due to adaptation to oligotrophic conditions, elevated hydrostatic pressure, and limited nutrient availability [18,19,20]. Comparative genomic studies suggest that these environmental pressures contribute substantially to biosynthetic diversification and may promote the accumulation of cryptic biosynthetic pathways [19].

Marine sponge-associated *Streptomyces* constitute another important ecological group because marine sponges harbor dense microbial consortia involved in chemical defense and symbiotic interactions. Numerous sponge-associated actinomycetes produce cytotoxic metabolites, quorum sensing inhibitors, antibiofilm compounds, and antimicrobial agents believed to contribute to host defense mechanisms. Similarly, mangrove-derived *Streptomyces* have attracted considerable interest because mangrove ecosystems represent highly fluctuating interfaces between terrestrial and marine environments characterized by changing salinity, oxygen gradients, and redox conditions [8,9]. Such environmental variability appears to promote metabolic flexibility and chemical diversity.

Comparative genomic analyses have revealed substantial variation in biosynthetic gene cluster content among marine-derived *Streptomyces*, and some marine lineages may exhibit higher BGC counts than particular terrestrial counterparts [12,19,43]. However, BGC content varies substantially among strains and ecological niches, and higher predicted BGC abundance should not be interpreted as a universal characteristic of marine-derived *Streptomyces*.

Overall, marine and terrestrial *Streptomyces* share a broadly conserved biosynthetic framework, including type I and type II polyketide synthases, non-ribosomal peptide synthetases, ribosomally synthesized and post-translationally modified peptide pathways, terpene biosynthetic clusters, regulatory genes, resistance determinants, transport systems, and tailoring enzymes [3,19]. The principal distinction is therefore not the presence of unique biosynthetic systems in marine strains, but differences in the abundance, diversity, regulation, and ecological deployment of these clusters [8,12,19]. Marine *Streptomyces* are exposed to environmental conditions such as salinity, hydrostatic pressure, nutrient limitation, oxygen and redox gradients, and intense microbial competition, which may favor expansion or diversification of secondary-metabolite pathways and accumulation of cryptic biosynthetic gene clusters [8,18,19]. In contrast, terrestrial *Streptomyces* have been extensively investigated in soil and other terrestrial habitats and have historically provided many clinically important metabolites [3,5,19]. These observations support the view that marine adaptation can reshape biosynthetic potential while retaining the core enzymatic logic shared with terrestrial *Streptomyces* [3,8,19]. Importantly, the reported enrichment of BGCs in marine strains should not be interpreted as a universal property of all marine *Streptomyces*, because BGC content varies among strains and ecological niches [12,19].

## 4. Biosynthetic Gene Clusters in Marine Streptomyces

Secondary metabolite biosynthesis in marine *Streptomyces* is primarily encoded by biosynthetic gene clusters comprising structural biosynthetic genes, regulatory proteins, resistance determinants, transport systems, and diverse tailoring enzymes [21,22]. These clusters collectively orchestrate highly coordinated biosynthetic pathways responsible for the production of structurally complex secondary metabolites.

The major biosynthetic systems identified in marine *Streptomyces* include type I and type II polyketide synthases (PKSs), non-ribosomal peptide synthetases (NRPSs), PKS-NRPS hybrid systems, ribosomally synthesized and post-translationally modified peptide (RiPP) pathways, and terpene biosynthetic clusters [12,13,14,15,16,17]. These biosynthetic systems collectively contribute to the chemical diversity observed among marine-derived *Streptomyces*. Representative biosynthetic classes and characteristic metabolites are summarized in Table 1.

Polyketide synthases represent one of the major biosynthetic systems in marine-derived *Streptomyces* and generate structurally diverse metabolites through modular or iterative enzymatic assembly processes [23,24]. Type I PKS systems catalyze successive condensation reactions using malonyl-CoA and related extender units, generating structurally complex polyketide scaffolds. Marine-derived actinomycetes produce structurally complex polyketides such as marinomycins and abyssomicins, whereas this review focuses specifically on polyketides experimentally associated with marine-derived *Streptomyces*. exhibit highly complex structures and potent biological activities [25,26]. Type II PKS systems, which generally function through sets of discrete enzymes, also contribute to the biosynthesis of structurally diverse aromatic polyketides, including angucycline- and anthracycline-type metabolites. Structural diversification of polyketides is further amplified by tailoring reactions including methylation, hydroxylation, glycosylation, oxidation, and halogenation.

Non-ribosomal peptide synthetases synthesize peptides independently of the ribosome through thiotemplate biosynthesis involving adenylation, thiolation, and condensation domains [23,24]. Marine NRPS-derived metabolites frequently contain unusual amino acids, D-amino acids, heterocyclic residues, and N-methylated structures that contribute substantially to their structural diversity and biological activities. Cyclomarins and related marine peptides have demonstrated potent antitubercular and anticancer properties [27].

PKS-NRPS hybrid systems represent an important class of biosynthetic pathways in marine-derived *Streptomyces* and generate structurally sophisticated metabolites incorporating both polyketide- and peptide-derived structural features [24,27]. These pathways combine enzymatic modules or domains associated with both PKS and NRPS biosynthesis and can generate chemically complex metabolites with diverse biological activities.

RiPP biosynthetic pathways constitute another important class of marine biosynthetic systems. These pathways encode precursor peptides that subsequently undergo extensive post-translational modifications to generate mature bioactive metabolites [16,17]. Marine RiPPs, including thiopeptides and lantipeptides, frequently exhibit potent antibacterial activities against Gram-positive pathogens.

Terpene biosynthetic gene clusters also contribute to the chemical diversity of marine *Streptomyces*. These pathways encode terpene synthases together with associated biosynthetic and tailoring enzymes and can generate structurally diverse marine isoprenoid metabolites. The presence of terpene biosynthetic capacity further expands the chemical space accessible to marine *Streptomyces*.

Despite major advances in biosynthetic annotation, a substantial disconnect remains between computational biosynthetic prediction and experimental validation. Many predicted clusters remain functionally uncharacterized, and annotation accuracy is frequently limited by incomplete understanding of marine biosynthetic enzymology. Consequently, excessive reliance on predictive bioinformatics without biochemical confirmation may contribute to annotation overinterpretation and inaccurate functional assignment.

To avoid overinterpretation of biosynthetic assignments, the examples discussed in this review are considered according to their level of supporting evidence. Experimentally characterized biosynthetic pathways are distinguished from genetically supported assignments, metabolomics-supported predictions, and purely bioinformatic predictions. Accordingly, the presence of a predicted BGC is interpreted as evidence of biosynthetic potential rather than confirmed metabolite production unless supported by appropriate genetic, biochemical, heterologous-expression, or metabolomics evidence [12,13,14,15,16,17,19,20].

## 5. Genome Mining Approaches

The emergence of affordable next-generation sequencing technologies has dramatically transformed marine natural product discovery by enabling systematic exploration of biosynthetic gene clusters at the genomic level [12,13]. Genome mining refers to the computational identification and prediction of secondary metabolite pathways based on conserved enzymatic motifs, domain organization, regulatory signatures, and comparative genomic architectures. This approach has become particularly important in research on marine-derived *Streptomyces* because conventional cultivation-based screening frequently fails to activate many of the biosynthetic pathways encoded within their genomes.

Importantly, genome-based prediction does not necessarily establish the production or biosynthetic function of the corresponding metabolite. In this review, biosynthetic assignments are therefore considered according to four levels of evidence: (i) experimentally characterized pathways, supported by direct genetic, biochemical, and/or heterologous-expression evidence; (ii) genetically supported assignments, in which genetic evidence supports a proposed biosynthetic function but does not establish the complete pathway; (iii) metabolomics-supported predictions, in which metabolite profiling, MS/MS analysis, molecular networking, or related approaches provide evidence supporting a proposed BGC–metabolite association; and (iv) purely bioinformatic predictions, in which BGCs or biosynthetic functions are inferred primarily from genome-mining algorithms, sequence homology, domain organization, or comparative genomics without direct experimental validation [12,13,14,15,16,17,19,20]. Throughout Section 4 and Section 5, predicted or putative pathways are described accordingly to distinguish biosynthetic potential from experimentally demonstrated biosynthetic function.

Several bioinformatic platforms have become central to marine biosynthetic discovery. antiSMASH is currently one of the most widely utilized tools for automated identification and annotation of biosynthetic gene clusters because it enables rapid recognition of PKS, NRPS, RiPP, terpene, and hybrid biosynthetic systems [13]. PRISM further enables predictive structural analysis of secondary metabolites based on biosynthetic domain organization and substrate specificity [12]. BAGEL specializes in the identification of RiPP-associated pathways and bacteriocin biosynthesis [16], whereas DeepBGC utilizes machine learning-assisted algorithms for improved biosynthetic cluster recognition [17]. BiG-SCAPE enables comparative clustering and evolutionary analysis of biosynthetic pathways across large genomic datasets, thereby facilitating biosynthetic family classification and diversity analysis [17].

Comparative genomic analyses have revealed substantial variation in biosynthetic gene cluster (BGC) content among marine-derived *Streptomyces*. For example, analysis of 87 marine *Streptomyces* genomes identified 16–84 secondary-metabolism BGCs per genome, indicating considerable strain- and ecotype-level variation in biosynthetic potential [43]. Nevertheless, only a subset of these predicted clusters is typically associated with metabolites detectable under standard laboratory cultivation conditions [14,15]. This discrepancy between biosynthetic potential and observable metabolite production has become one of the central conceptual challenges in marine natural product research. Consequently, activation of silent or cryptic biosynthetic pathways has become a major research priority.

To overcome transcriptional silencing, several activation strategies have been developed including OSMAC cultivation systems, co-cultivation approaches, ribosome engineering, epigenetic modulation, CRISPR-Cas-mediated activation, and heterologous expression platforms [14,15]. OSMAC strategies manipulate environmental parameters such as salinity, nutrient composition, pH, aeration, trace elements, and temperature in order to induce alternative metabolic states. Co-cultivation systems exploit microbial interactions and chemical signaling mechanisms to stimulate cryptic biosynthesis that may not occur in monoculture conditions. Ribosome engineering introduces mutations affecting translational regulation and secondary metabolism, whereas CRISPR-based approaches allow direct transcriptional activation of silent gene clusters through targeted promoter engineering and regulatory rewiring [15].

The rapid expansion of genome mining technologies has substantially accelerated biosynthetic pathway prediction and natural-product prioritization in marine-derived *Streptomyces*. Major bioinformatic platforms currently used in marine natural product discovery are summarized in Table 2.

Genome mining has substantially expanded access to previously unrecognized biosynthetic diversity; however, computational identification of a BGC does not by itself establish metabolite production or pathway function. The principal limitation of genome-based discovery therefore lies in distinguishing biosynthetic potential from experimentally demonstrated activity. Accordingly, genome mining is most informative when integrated with metabolomics, transcriptomics, and targeted genetic or biochemical validation to prioritize BGCs for functional investigation [12,13,14,15,16,17,19,20].

## 6. Biosynthetic Enzymology and Tailoring Reactions

One characteristic of natural products produced by marine-derived *Streptomyces* is the diversity of enzymatic tailoring reactions that transform core biosynthetic scaffolds into structurally complex metabolites. Tailoring enzymes contribute to chemical diversification by modifying the structure, stereochemistry, polarity, and other physicochemical properties of biosynthetic intermediates. In many cases, the biological properties of marine metabolites depend not only on their core biosynthetic scaffolds but also on post-assembly enzymatic modifications that can influence molecular recognition, stability, solubility, and other compound-specific properties.

Halogenation represents an important biosynthetic modification in some marine-derived *Streptomyces* pathways, where enzymatic halogenation can introduce chlorine or bromine into specialized metabolites [28,29,30,31]. Some marine-derived *Streptomyces* encode flavin-dependent halogenases, non-heme iron halogenases, and vanadium-dependent haloperoxidases capable of catalyzing regioselective chlorination and bromination reactions [28,29,30,31]. Halogenated marine metabolites, including marinopyrroles and napyradiomycins, illustrate how halogen incorporation can contribute to structural diversification; however, the effects of halogenation on potency, stability, permeability, and other biological properties are compound- and target-dependent [32,33]. The discovery of selective marine halogenases has therefore attracted interest in biocatalysis and synthetic biology because these enzymes provide useful biocatalytic routes for introducing halogen substituents into complex molecular scaffolds.

Glycosylation represents another major tailoring strategy in marine biosynthesis. Glycosyltransferases attach deoxysugars and modified carbohydrate residues to aglycone scaffolds, thereby generating structural diversity and potentially modifying properties such as solubility, molecular recognition, and pharmacokinetic behavior [34,35]. The biological consequences of glycosylation are highly dependent on the aglycone, sugar moiety, attachment site, and molecular target. Marine metabolites such as lobophorins and kijanimicin analogs contain highly complex glycosylated structures generated through specialized sugar biosynthetic cassettes embedded within biosynthetic gene clusters [27,36]. Thus, glycosylation should be considered an important source of structural diversification rather than a universal determinant of improved pharmacological activity.

Cytochrome P450 monooxygenases are also abundant in marine-derived *Streptomyces* genomes and catalyze oxidation, hydroxylation, epoxidation, and oxidative cyclization reactions that can increase molecular complexity and stereochemical diversity [37,38]. Sequential oxidative tailoring can substantially influence the structural and physicochemical properties of marine metabolites, although the biological consequences depend on the specific scaffold and modification.

Macrocyclization can contribute to conformational constraint in marine natural products. Thioesterases, cyclases, and Diels–Alderases participate in ring-closure and cyclization reactions that generate macrocycles, polyethers, and spirotetronate architectures [39]. Enzymatic Diels–Alder reactions can generate complex polycyclic frameworks with defined stereochemical configurations under enzymatic conditions; however, the contribution of individual structural features to biological activity or molecular recognition is compound-specific.

Despite major advances in biosynthetic enzymology and structural prediction, many marine tailoring enzymes remain poorly characterized at the biochemical and mechanistic levels. Enzyme functions are frequently inferred from sequence homology or domain architecture without direct biochemical confirmation, creating a risk of functional overinterpretation [29,30,31,37,38,39]. Resolving this limitation will require closer integration of structural biology, enzymology, metabolomics, and synthetic biology to establish experimentally supported functions and to exploit marine biosynthetic enzymes as potential biocatalysts.

## 7. Polyketide Diversity in Marine Streptomyces

Polyketides constitute one of the major classes of structurally diverse natural products produced by marine-derived *Streptomyces* and are biosynthesized through successive decarboxylative Claisen condensation reactions catalyzed by polyketide synthases (PKSs) [40,41]. Marine-derived *Streptomyces* frequently produce polyketides with complex architectures, including highly unsaturated macrocycles, polyenes, aromatic scaffolds, spirotetronates, and macrodiolides. This structural diversity arises from the modular organization of PKS assembly systems and extensive post-assembly tailoring reactions, which together expand the chemical space accessible to marine-derived *Streptomyces*.

Type I PKSs are large multifunctional enzymes that generally contain multiple catalytic domains organized into modules, with each module typically contributing to one cycle of chain extension and processing [40]. Variation in ketosynthase, acyltransferase, ketoreductase, dehydratase, and enoylreductase activities provides substantial control over carbon-chain extension, reduction state, and stereochemical configuration. These modular biosynthetic systems contribute to the formation of structurally complex marine polyketides, particularly metabolites containing highly substituted macrocycles, polyenes, and multiple stereogenic centers.

Representative polyketides reported from marine-derived *Streptomyces* include saliniketals, marinomycins, arenicolides, abyssomicins, and lobophorins [41,42]. Marinomycins are highly unsaturated macrodiolide polyketides that exhibit notable cytotoxic and antimicrobial activities and are characterized by complex PKS-derived architectures [42]. Abyssomicins constitute a distinct family of sulfur-containing and polycyclic polyketides, among which abyssomicin C is particularly notable for its inhibition of para-aminobenzoic acid (PABA) biosynthesis through targeting of the chorismate pathway, contributing to its antibacterial activity. Lobophorins are glycosylated spirotetronate polyketides that display antibacterial and cytotoxic activities associated with their highly modified macrocyclic scaffolds.

Type II PKSs differ from the modular Type I systems because they are composed of discrete, dissociated enzymes that act iteratively during polyketide chain assembly [40,41]. These systems are particularly important for the biosynthesis of aromatic polyketides, including angucyclines, tetracenomycins, and related aromatic metabolites. Marine-derived *Streptomyces* possessing Type II PKS biosynthetic gene clusters can therefore generate structurally diverse aromatic compounds with antibacterial, cytotoxic, kinase-inhibitory, and other pharmacological activities. Further structural diversification is achieved through tailoring reactions such as oxidation, glycosylation, methylation, halogenation, and cyclization.

Spirotetronates represent an important subclass of polyketides reported from marine-derived *Streptomyces* and other marine-associated actinomycetes. These metabolites contain a tetronic acid-derived spirocyclic or spirotetronate motif incorporated into complex macrocyclic scaffolds and often exhibit highly constrained three-dimensional architectures. Representative examples include lobophorins and chlorothricin, which display antibacterial and/or cytotoxic activities [41,42]. Their biological properties are influenced by the combination of rigid macrocyclic frameworks, glycosylation patterns, and extensive stereochemical complexity. In contrast, abyssomicins should be considered a distinct class of polycyclic polyketides rather than grouped with spirotetronates.

The structural diversity of marine polyketides is further enhanced by tailoring reactions that occur during or after assembly of the core carbon skeleton. Glycosylation, methylation, oxidation, epoxidation, cyclization, and halogenation can substantially modify physicochemical properties and biological activities. In particular, halogenating enzymes encoded within some marine-derived biosynthetic gene clusters can introduce chlorine or bromine into specialized metabolites, potentially contributing to increased structural diversity and biological activity. However, halogenation is a biosynthetic feature of specific pathways rather than a universal characteristic of marine polyketides.

Despite their substantial chemical diversity and pharmacological potential, translation of marine polyketides into clinically applicable therapeutics remains limited by several major challenges. Many metabolites are produced at low concentrations under laboratory cultivation conditions, while silent or poorly expressed biosynthetic gene clusters can limit their discovery and production. In addition, the structural complexity and extensive stereochemistry of many marine polyketides can complicate purification, fermentation scale-up, structural optimization, and total or semisynthetic production. Consequently, future development of marine polyketide-based therapeutics will require integrated approaches combining genome mining, biosynthetic gene-cluster activation, pathway engineering, heterologous expression, scalable fermentation, and synthetic biology. The major classes of marine-derived polyketides and their reported biological activities are summarized in Table 3.

## 8. Non-Ribosomal Peptides

Non-ribosomal peptides (NRPs) are synthesized by large modular non-ribosomal peptide synthetase (NRPS) megaenzymes through thiotemplate mechanisms independent of the ribosome [51]. Each NRPS module typically contains adenylation, thiolation, and condensation domains responsible for amino acid activation, carrier attachment, and peptide bond formation. Unlike ribosomal biosynthesis, NRPS systems are capable of incorporating highly unusual amino acids, hydroxy acids, D-amino acids, heterocyclic residues, and N-methylated building blocks, thereby generating substantial structural diversity and chemically complex metabolites.

Marine *Streptomyces* produce structurally distinctive non-ribosomal peptides enriched with rare amino acid residues and highly modified peptide architectures. These metabolites have been reported to exhibit diverse biological activities, including antibacterial, antifungal, anticancer, antiparasitic, and anti-inflammatory effects. Their structural and chemical diversity may contribute to interactions with a range of biological targets, although the mechanisms and structure–activity relationships are compound-specific.

Cyclomarins are cyclic heptapeptides originally isolated from the marine-derived *Streptomyces* sp. CNB-982 [44]. Cyclomarin A specifically targets the ClpC1 subunit of the caseinolytic protease system in *Mycobacterium tuberculosis*, providing a mechanistically distinct target for antitubercular drug discovery. Structural and genetic studies have established direct interaction with ClpC1 and demonstrated that alterations in the ClpC1 target site can confer resistance, thereby providing experimental support for its mechanism of action [52]. Importantly, subsequent medicinal-chemistry studies have generated synthetic cyclomarin derivatives and evaluated their structure–activity relationships, demonstrating that chemical optimization of this marine natural-product scaffold can produce analogues with improved or differentiated biological properties [53,54]. These studies illustrate a translational progression from natural-product discovery and target identification to mechanism-of-action studies and lead optimization.

Ohmyungsamycins A and B are cyclic depsipeptides isolated from a marine-derived *Streptomyces* strain collected from a sand beach on Jeju Island, Korea [48].

Marine NRPS-derived metabolites also display substantial structural diversity through hybridization with PKS systems. PKS-NRPS hybrid pathways integrate peptide and polyketide biosynthetic modules, generating highly sophisticated metabolites with exceptional stereochemical complexity [40,41]. These hybrid compounds have demonstrated diverse biological activities in experimental studies, including cytotoxic and antiparasitic activities.

Despite their pharmacological potential, marine non-ribosomal peptides present several challenges for translational development, including structural complexity, limited stability, poor oral bioavailability, and difficulties associated with pathway engineering and production. These features highlight the need for improved metabolic engineering, heterologous expression, and chemical optimization strategies to enhance the accessibility and developability of marine NRPS-derived metabolites. The major classes of marine non-ribosomal peptides are summarized in Table 4.

The extraordinary structural diversity of marine non-ribosomal peptides continues to provide valuable opportunities for antibiotic discovery. However, many studies remain limited to initial bioactivity screening without elucidating molecular targets or mechanisms of action. Future investigations should increasingly incorporate target-based screening, chemical biology, and systems pharmacology approaches to improve translational relevance [24,27,36,40,41,42].

## 9. Ribosomally Synthesized and Post-Translationally Modified Peptides (RiPPs)

Genomic analyses of marine-derived *Streptomyces* have identified diverse predicted RiPP biosynthetic gene clusters, including pathways associated with lantipeptides, lasso peptides, thiopeptides, and other peptide-derived metabolites [16,17]. Unlike NRPS-derived metabolites, RiPP biosynthesis begins with ribosomal peptide synthesis followed by highly specialized post-translational tailoring reactions including cyclization, dehydration, methylation, epimerization, heterocyclization, and proteolytic processing. These modifications collectively generate structurally complex metabolites with diverse biological activities.

Marine *Streptomyces* genomes contain numerous cryptic RiPP biosynthetic gene clusters encoding lantipeptides, lasso peptides, thiopeptides, cyanobactin-like compounds, and other peptide-derived metabolites [16,17]. These findings indicate that marine-derived *Streptomyces* possess substantial predicted RiPP biosynthetic potential, although many predicted pathways remain experimentally uncharacterized, suggesting that marine ecosystems remain an underexplored source of peptide diversity.

One major advantage of RiPP biosynthesis is its genetic simplicity relative to PKS and NRPS systems. Because precursor peptides are directly encoded by structural genes, RiPP pathways are often more amenable to genetic engineering, combinatorial biosynthesis, and synthetic biology-based diversification. This modularity has attracted increasing interest for rational peptide engineering and next-generation antimicrobial development.

Thiopeptides isolated from marine actinomycetes exhibit potent antibacterial activity in reported experimental studies against Gram-positive pathogens and have emerged as promising candidates for antimicrobial drug discovery [16]. These sulfur-rich macrocyclic peptides frequently target ribosomal machinery or essential translational processes, thereby providing highly selective antibacterial activity. Similarly, lantipeptides possess unusual lanthionine bridges generated through post-translational dehydration and cyclization reactions, contributing to enhanced structural rigidity and proteolytic stability.

Lasso peptides represent another distinctive RiPP class characterized by threaded peptide topologies resembling molecular knots. Their conformational stability frequently confers resistance to thermal degradation and proteolysis, making them particularly attractive for pharmaceutical applications. Marine-derived RiPPs may therefore represent valuable scaffolds for development of peptide-based therapeutics with improved stability and target specificity.

Despite increasing genomic evidence for RiPP diversity in marine microorganisms, many predicted RiPP pathways remain experimentally uncharacterized [16,17]. In numerous cases, computational prediction has substantially outpaced biochemical validation. Consequently, a major conceptual gap remains between genomic biosynthetic prediction and experimentally verified metabolite production. Furthermore, low expression levels, cryptic regulation, and insufficient cultivation strategies continue to limit access to many marine RiPP metabolites.

Another important limitation involves annotation accuracy. Automated genome mining algorithms frequently predict RiPP clusters based solely on conserved motifs and precursor peptide signatures, which may result in false-positive predictions or incomplete pathway annotation. Therefore, future progress in marine RiPP research will require stronger integration of comparative genomics, structural biology, metabolomics, and functional enzymology to achieve experimentally validated understanding of marine peptide biosynthesis. Major classes of marine RiPPs and their biosynthetic features are summarized in Table 5.

## 10. Terpenoids and Isoprenoid Metabolites

Although marine actinomycete research has historically focused predominantly on polyketides and non-ribosomal peptides, terpenoid biosynthesis is increasingly recognized as an important metabolic capability in marine *Streptomyces* [18,20]. Terpenoids constitute one of the largest classes of natural products in nature and are biosynthesized through condensation of isoprene-derived precursors generated via either the mevalonate pathway or the methylerythritol phosphate (MEP) pathway.

Marine-derived terpenoids frequently exhibit cytotoxic, antimicrobial, antioxidant, antiviral, and anti-inflammatory activities. In contrast to many terrestrial terpenoids, some terpenoids reported from marine-derived *Streptomyces* possess rearranged carbon skeletons, highly oxidized frameworks, or other unusual structural features [28,29,30,31]. These structural features contribute significantly to biological activity and pharmacological specificity.

Marine *Streptomyces* genomes encode diverse terpene cyclases and prenyltransferases capable of generating structurally complex mono-, sesqui-, di-, and triterpenoid metabolites. Biosynthetic diversification is further enhanced through oxidative tailoring reactions mediated by cytochrome P450 monooxygenases and related oxidative enzymes [37,38]. Such enzymatic modifications generate highly modified terpenoid scaffolds rarely observed in terrestrial microorganisms.

Several marine terpenoids possess unusual carbocyclic frameworks resulting from extensive rearrangement reactions during biosynthesis. These structural rearrangements frequently produce metabolites with conformational constraints and potentially altered molecular recognition. Additionally, marine terpenoids may contribute ecologically to chemical defense, microbial competition, signaling interactions, and environmental adaptation within marine microbial communities.

Despite growing recognition of marine terpenoid diversity, this biosynthetic field remains comparatively underexplored relative to PKS- and NRPS-derived metabolites. One major reason is that terpene biosynthetic pathways are frequently smaller and more difficult to identify computationally using conventional genome mining algorithms. Furthermore, many marine terpenoids are produced at low concentrations, complicating metabolite isolation and structural characterization.

Another important limitation involves insufficient functional characterization of marine terpene cyclases and tailoring enzymes. Many predicted terpene biosynthetic clusters remain experimentally unvalidated, and mechanistic understanding of marine terpene biosynthesis remains incomplete. Consequently, future research integrating genome mining, structural biology, enzymology, and metabolomics will be essential for expanding understanding of marine terpenoid biosynthesis and pharmaceutical potential.

The major classes of marine terpenoids and their biosynthetic characteristics are summarized in Table 6.

Compared with polyketides and non-ribosomal peptides, marine terpenoids remain comparatively understudied, and their biosynthetic diversity is likely underestimated. This imbalance may reflect methodological limitations in detecting terpene biosynthetic pathways and the limited characterization of marine terpene cyclases. Greater characterization of these enzymes and their associated pathways may therefore reveal additional terpenoid scaffolds with ecological and pharmacological relevance [18,19,20,28,29,30,31,37,38].

## 11. Metabolomics and Dereplication Strategies

Modern marine natural product discovery increasingly relies on metabolomics-guided workflows integrating LC-MS/MS, UHPLC-QTOF, NMR metabolomics, imaging mass spectrometry, and molecular networking approaches [16,17]. These technologies have substantially expanded the analytical capacity of marine biosynthetic research by enabling high-throughput chemical profiling and rapid prioritization of structurally novel metabolites from highly complex microbial extracts.

Dereplication refers to the rapid identification of previously known compounds during early stages of natural product screening in order to minimize repetitive rediscovery of structurally characterized metabolites [16]. Historically, marine natural product discovery suffered from substantial inefficiency because many studies repeatedly isolated already known antibiotics or cytotoxic compounds. Metabolomics-guided dereplication strategies now allow researchers to identify known metabolites at early analytical stages, thereby improving discovery efficiency and prioritization of chemically unique compounds.

GNPS molecular networking has emerged as one of the most valuable platforms in contemporary natural product metabolomics because it enables clustering of structurally related metabolites based on MS/MS fragmentation similarity [16]. This approach facilitates visualization of chemical families and allows rapid identification of analog series, biosynthetic relationships, and potentially novel metabolites within complex datasets. Molecular networking has proven particularly valuable for marine *Streptomyces* extracts because these organisms frequently produce diverse families of structurally related secondary metabolites.

Untargeted metabolomics further enables comparative analysis of metabolic responses under different cultivation conditions including OSMAC systems, co-cultivation strategies, salinity variation, and nutrient modulation. Such approaches can reveal cryptic metabolites that remain undetected under conventional cultivation conditions. Imaging mass spectrometry additionally enables spatial visualization of metabolite distribution during microbial interactions, thereby providing insight into ecological functions of marine secondary metabolites.

NMR-based metabolomics also contributes significantly to marine natural product discovery because it enables structural characterization of metabolites without complete purification. Combined LC-MS/MS and NMR approaches therefore provide complementary analytical platforms for structural elucidation and biosynthetic interpretation.

Despite substantial technological advances, metabolomics-guided discovery remains constrained by incomplete spectral databases, low-abundance metabolites, ion suppression, extraction biases, and uncertainty associated with automated annotation [16,17]. Modern platforms can generate thousands of molecular features from a single extract, yet only a fraction can be confidently assigned, structurally characterized, or biologically validated. These limitations highlight the need to improve spectral reference databases, annotation confidence, metabolite isolation, and orthogonal structural and functional validation rather than relying solely on computational feature annotation [12,16,17,20].

The major metabolomics technologies currently applied in marine natural product discovery are summarized in Table 7.

## 12. Synthetic Biology and Drug Development

Synthetic biology has emerged as an important strategy for engineering marine natural product biosynthesis and overcoming many limitations associated with conventional cultivation-based metabolite discovery [14,15]. Advances in genome engineering, pathway refactoring, heterologous expression, chassis optimization, and CRISPR-Cas technologies now enable targeted manipulation of marine biosynthetic pathways for enhanced metabolite production and diversification.

One of the major applications of synthetic biology in marine actinomycete research involves activation of silent or cryptic biosynthetic gene clusters [14,15]. Many marine *Streptomyces* genomes contain numerous biosynthetic pathways that remain transcriptionally inactive under standard laboratory conditions. Synthetic biology approaches allow targeted activation of these pathways through promoter engineering, transcriptional rewiring, regulatory manipulation, and heterologous pathway reconstruction.

Engineered hosts such as *Streptomyces coelicolor* and *Streptomyces albus* are widely utilized as heterologous expression platforms because they possess well-characterized genetic systems and relatively low endogenous metabolite backgrounds [4,15]. Heterologous expression enables production of marine metabolites in genetically tractable hosts capable of improved fermentation performance and biosynthetic accessibility.

CRISPR-Cas systems have further expanded the capabilities of marine biosynthetic engineering by enabling precise genome editing and transcriptional regulation [15]. CRISPR-based activation systems can directly stimulate silent biosynthetic clusters, whereas multiplex editing approaches enable simultaneous manipulation of multiple biosynthetic pathways. Such technologies provide opportunities for combinatorial biosynthesis and generation of structurally diversified metabolite libraries.

Synthetic biology also enables pathway refactoring through modular reconstruction of biosynthetic systems independent of native regulatory constraints. Refactored pathways can be optimized for improved precursor supply, enhanced metabolic flux, and increased production yield. Additionally, artificial intelligence-assisted pathway prediction and machine learning-guided metabolite prioritization are increasingly integrated into synthetic biology workflows to accelerate rational natural product engineering.

Marine-derived actinomycetes have demonstrated substantial pharmaceutical potential in oncology, infectious disease, inflammation, neurodegeneration, and metabolic disorders [1,11,42]. Although salinosporamide A (marizomib) is not a *Streptomyces*-derived metabolite, it provides an informative comparative example of the translational potential of marine actinomycetes. Salinosporamide A is a potent proteasome inhibitor produced by the obligate marine actinomycete *Salinispora tropica* [55,56]. Mechanistic studies established its irreversible inhibition of the 20S proteasome, while preclinical studies demonstrated activity in multiple hematological and solid-tumor models [55,56]. Medicinal-chemistry and structure–activity relationship studies subsequently generated and evaluated salinosporamide analogues, illustrating how structural modification can be used to investigate pharmacological activity and optimize marine natural-product scaffolds [57].

Importantly, salinosporamide A progressed from marine natural-product discovery through preclinical evaluation and medicinal-chemistry development into human clinical trials, providing a well-documented example of the translational pathway from marine microbial biosynthesis to drug development [55,56]. Its development also involved complementary production strategies, including fermentation, precursor-directed biosynthesis, mutasynthesis, semisynthesis, and total synthesis, highlighting the importance of scalable and chemically flexible approaches for advancing structurally complex marine natural products toward pharmaceutical development [58].

Nevertheless, successful pharmaceutical development requires more than efficient pathway engineering. Marine natural products may face limitations related to toxicity, stability, pharmacokinetic behavior, bioavailability, and manufacturing feasibility. These considerations emphasize the importance of integrating biosynthetic engineering with downstream pharmacological and process-development strategies.

The major synthetic biology strategies currently applied in marine natural product engineering are summarized in Table 8.

While synthetic biology offers substantial opportunities for pathway engineering and metabolite diversification, successful translation requires careful consideration of industrial feasibility. Engineering increasingly complex biosynthetic systems may improve chemical diversity but can simultaneously impose significant metabolic burdens on production hosts. Balancing innovation with manufacturability will therefore remain a central challenge for future marine natural product development [4,14,15,17].

## 13. Challenges and Future Perspectives

Despite substantial technological progress, marine natural product discovery continues to face several major scientific, methodological, and translational limitations that collectively restrict efficient pharmaceutical development [12,13,14,15,16,17]. Although genome mining, metabolomics, synthetic biology, and artificial intelligence-assisted discovery platforms have accelerated biosynthetic exploration, many fundamental bottlenecks remain unresolved.

One of the most persistent limitations involves the low cultivation efficiency of marine microorganisms. A large proportion of marine microbial diversity remains uncultivable under conventional laboratory conditions because native marine ecosystems possess highly specific physicochemical and ecological parameters that are difficult to reproduce experimentally [18,19,20]. Consequently, many biosynthetic pathways encoded within marine microbial genomes remain inaccessible for experimental characterization. Even cultivable strains frequently display unstable metabolite production due to environmental sensitivity, nutritional dependence, or complex microbial interactions.

Silent biosynthetic gene clusters represent another major challenge in marine-derived *Streptomyces* research [14,15]. Genome sequencing studies have demonstrated that marine-derived *Streptomyces* can harbor substantial biosynthetic potential that is not necessarily reflected by observable metabolite production. However, activation of cryptic pathways remains highly unpredictable, and many activation strategies produce only trace quantities of metabolites insufficient for structural elucidation or pharmacological testing. Furthermore, heterologous expression systems often fail to reproduce native regulatory networks required for complete pathway functionality.

Rediscovery of structurally known metabolites also remains a substantial obstacle despite major advances in dereplication technologies [16]. Marine microorganisms frequently produce common biosynthetic scaffolds shared across multiple taxa, thereby reducing discovery efficiency. Although metabolomics-guided prioritization and GNPS molecular networking have improved dereplication workflows, repetitive isolation of previously characterized compounds continues to consume considerable research resources.

Another important challenge involves annotation overinterpretation arising from excessive dependence on computational prediction [12,17]. Artificial intelligence-assisted genome mining and machine learning-based biosynthetic analysis have greatly expanded biosynthetic prediction capacity, but computational annotations frequently lack biochemical validation. Many predicted biosynthetic clusters are assigned hypothetical functions solely through sequence homology without experimental confirmation. Consequently, the field increasingly faces risks associated with annotation inflation, metabolite misidentification, and overestimation of biosynthetic novelty.

Industrial scalability also remains a major translational limitation. Numerous marine metabolites exhibit highly promising biological activities but cannot be efficiently produced at industrial scale because of low biosynthetic yield, metabolic instability, or highly complex structural features. In addition, many marine-derived compounds possess poor pharmacokinetic profiles, limited aqueous solubility, or unacceptable toxicity, thereby restricting clinical applicability despite impressive in vitro bioactivity.

Another conceptual limitation involves insufficient ecological interpretation of marine secondary metabolism. Many current studies emphasize metabolite identification and biosynthetic prediction without adequately investigating ecological function, microbial interactions, evolutionary significance, or environmental regulation. Secondary metabolites likely play important ecological roles involving competition, signaling, symbiosis, defense, and environmental adaptation within marine microbial communities. However, these ecological dimensions remain comparatively underexplored relative to purely chemical characterization.

Future progress in marine *Streptomyces* research will likely depend on stronger integration of long-read sequencing, single-cell genomics, spatial metabolomics, systems biology, artificial intelligence-assisted genome mining, synthetic ecology, automated biofoundry systems, and high-throughput functional screening [12,13,14,15,16,17]. Long-read sequencing technologies may substantially improve biosynthetic gene cluster assembly and structural accuracy, whereas single-cell genomics may provide access to previously uncultivable microbial populations.

Spatial metabolomics and imaging mass spectrometry may further improve understanding of ecological interactions and metabolite localization within marine microbial communities. Simultaneously, synthetic ecology approaches involving controlled multispecies cultivation systems may better reproduce natural microbial signaling interactions responsible for cryptic metabolite activation.

Artificial intelligence-assisted biosynthetic prediction will likely continue to accelerate marine natural product discovery, but future research must balance computational prediction with rigorous experimental validation. Functional genomics, structural enzymology, metabolomics, and translational pharmacology should therefore become more tightly integrated to ensure biologically meaningful interpretation of biosynthetic datasets.

Major translational challenges and potential future solutions in marine *Streptomyces* drug discovery are summarized in Table 9.

Perhaps the most important conceptual transition facing the field is the movement from discovery-driven research toward function-driven biosynthetic biology. Future studies should not only ask what metabolites are produced but also why they are produced, how they function within natural ecosystems, and whether they possess realistic translational potential. Addressing these questions will require stronger integration between microbial ecology, functional genomics, metabolomics, and pharmacology [12,13,14,15,16,17,18,19,20].

## 14. Conclusions

Marine-derived *Streptomyces* represent an important source of structurally diverse and pharmacologically relevant natural products. Their biosynthetic capacity encompasses polyketides, non-ribosomal peptides, RiPPs, terpenoids, alkaloids, and hybrid metabolites with diverse biological activities [1,2,3].

Recent advances in genome sequencing, genome mining, metabolomics, molecular networking, synthetic biology, and artificial intelligence-assisted discovery have substantially expanded our understanding of marine biosynthetic diversity [12,13,14,15,16,17]. These approaches have revealed substantial biosynthetic potential in marine-derived *Streptomyces*, including numerous predicted biosynthetic gene clusters (BGCs) that remain experimentally uncharacterized and may represent cryptic pathways that are not readily accessible under conventional cultivation conditions. At the same time, advances in biosynthetic enzymology have demonstrated how tailoring reactions, including halogenation, glycosylation, oxidation, cyclization, and macrocyclization, contribute to the structural and functional diversification of natural products produced by marine-derived *Streptomyces* [28,29,30,31,32,33,34,35,36,37,38,39].

Despite this technological progress, an important distinction remains between predicted biosynthetic potential and experimentally demonstrated biosynthetic function. Many predicted BGCs remain functionally uncharacterized, while metabolite production, biological function, and translational feasibility cannot be established from computational prediction alone. This highlights the importance of experimental validation and careful interpretation of biosynthetic assignments throughout marine natural-product research.

Future progress will therefore require integrated workflows that connect genome mining with metabolomics, functional genomics, mechanistic enzymology, medicinal chemistry, synthetic biology, scalable production, ecological investigation, and translational pharmacology.

The major strength of this review lies in its integrative and critical perspective, linking marine microbial ecology, biosynthetic genomics, metabolomics, enzymology, synthetic biology, and translational pharmacology within a unified framework. The future impact of marine natural-product research will ultimately depend not only on the discovery of new biosynthetic diversity but also on the ability to translate this knowledge into experimentally validated biological functions, clinically relevant therapeutics, and sustainable biotechnological applications. Marine-derived *Streptomyces* therefore remain an incompletely explored reservoir whose full pharmaceutical and biotechnological potential will depend on interdisciplinary strategies that connect biosynthetic prediction with functional validation and translational development.

## Figures and Tables

**Table 1:** Major Biosynthetic Classes Identified in Marine-Derived *Streptomyces*.

Biosynthetic Class	Representative Metabolites	Major Biological Activities	Key Biosynthetic Features	References
Type I polyketides	Marinopyrroles	Antibacterial	Modular/iterative PKS-mediated biosynthesis with extensive tailoring	[26]
Type II polyketides	Angucyclines, anthracycline-type metabolites	Antibacterial, cytotoxic	Aromatic Type II PKS systems	[23,24]
Non-ribosomal peptides	Cyclomarins, ohmyungsamycins	Antitubercular, anticancer	NRPS thiotemplate biosynthesis	[24,27]
RiPPs	Thiopeptides, lantipeptides	Antibacterial	Ribosomal precursor synthesis followed by post-translational modification	[16,17]
Terpenoids	Marine *Streptomyces* terpenoids	Antioxidant, antimicrobial	Terpene cyclases and associated tailoring enzymes	[18,19,20]

**Table 2:** Major Genome Mining and Bioinformatic Platforms Used in Marine Natural Product Discovery.

Platform	Primary Application	Major Strengths	Current Limitations	References
antiSMASH	Automated BGC annotation	Comprehensive detection	Limited novel enzyme prediction	[13]
PRISM	Metabolite structure prediction	Predictive biosynthetic modeling	Dependent on known pathways	[12]
BAGEL	RiPP identification	Specialized RiPP analysis	Limited non-RiPP analysis	[16]
DeepBGC	Machine learning-assisted prediction	Improved sensitivity	Training dataset dependency	[17]
BiG-SCAPE	Comparative BGC clustering	Evolutionary analysis	Functional validation required	[17]

**Table 3:** Representative Polyketides Produced by Marine-Derived *Streptomyces*.

Polyketide Class	Representative Metabolites	Major Biological Activities	Key Structural Features	References
Macrodiolides	Marinomycins	Anticancer, antibacterial	Highly unsaturated macrodiolide architectures	[42]
Spirotetronates	Lobophorins, chlorothricin	Antibacterial, cytotoxic	Tetronic acid-containing spirocyclic macrocycles; glycosylated scaffolds	[41,42]
Polycyclic polyketides	Abyssomicins	Antibacterial	Highly modified polycyclic scaffolds	[41,42]
Aromatic polyketides	Anthracyclines, angucyclines, tetracenomycins	Anticancer, antibacterial, kinase inhibition	Fused aromatic ring systems	[40,41]

**Table 4:** Representative Non-Ribosomal Peptides Produced by Marine-Derived *Streptomyces*.

Metabolite Class	Representative Compounds	Major Biological Activities	Key Structural Features	References
Cyclic peptides	Cyclomarins	Antitubercular, anti-inflammatory	Cyclic heptapeptides	[36,42]
Cyclic depsipeptides	Ohmyungsamycins	Antibacterial, anticancer	Ester-linked peptide macrocycles	[36]

**Table 5:** Major RiPP Classes Identified in Marine-Derived *Streptomyces*.

RiPP Class	Representative Features	Major Biological Activities	Key Biosynthetic Modifications	References
Thiopeptides	Sulfur-rich macrocycles	Potent antibacterial activity	Cyclodehydration and thiazole formation	[16,17]
Lantipeptides	Lanthionine-containing peptides	Antimicrobial activity	Dehydration and thioether bridge formation	[16]
Lasso peptides	Threaded peptide topology	Proteolytic stability, antibacterial activity	Macrolactam ring threading	[17]
Cyanobactin-like peptides	Cyclic modified peptides	Cytotoxic and antimicrobial activity	Cyclization and heterocyclization	[16,17]

**Table 6:** Major Classes of Terpenoids Reported from Marine-Derived *Streptomyces*.

Terpenoid Class	Major Biological Activities	Key Biosynthetic Features	Structural Characteristics	References
Monoterpenoids	Antimicrobial activity	Isoprene condensation	Small volatile structures	[18,20]
Sesquiterpenoids	Cytotoxic, anti-inflammatory	Farnesyl pyrophosphate-derived	Rearranged carbon skeletons	[20]
Diterpenoids	Antioxidant, antimicrobial	Geranylgeranyl pyrophosphate-derived	Polycyclic frameworks	[18]
Halogenated terpenoids	Antibacterial, antiviral	Halogenase-associated pathways	Brominated/chlorinated scaffolds	[28,29,30,31]

**Table 7:** Major Metabolomics and Dereplication Technologies Used in Marine Natural Product Discovery.

Technology	Primary Application	Major Strengths	Current Limitations	References
LC-MS/MS	Metabolite profiling	High sensitivity	Ion suppression effects	[16]
UHPLC-QTOF	High-resolution metabolomics	Accurate mass detection	Complex data interpretation	[16,17]
GNPS molecular networking	Metabolite clustering	Rapid dereplication	Database dependency	[16]
NMR metabolomics	Structural elucidation	Non-destructive analysis	Lower sensitivity	[17]
Imaging mass spectrometry	Spatial metabolite visualization	Ecological interaction analysis	Expensive instrumentation	[16,17]

**Table 8:** Synthetic Biology Approaches for Marine-Derived *Streptomyces* Natural-Product Discovery and Development.

Synthetic Biology Strategy	Major Application	Key Advantages	Current Limitations	References
CRISPR-Cas activation	Silent BGC activation	Precise genome regulation	Off-target effects	[15]
Heterologous expression	Metabolite production	Improved biosynthetic accessibility	Host compatibility issues	[14,15]
Pathway refactoring	Biosynthetic optimization	Regulatory independence	Complex engineering requirements	[15]
Chassis engineering	Industrial metabolite production	Enhanced scalability	Metabolic burden	[14]
AI-assisted pathway prediction	Metabolite prioritization	Accelerated discovery	Annotation overprediction	[17]

**Table 9:** Major Translational Challenges in Marine-Derived *Streptomyces* Natural-Product Discovery and Development.

Challenge	Current Impact	Potential Future Solutions	References
Silent biosynthetic gene clusters	Limited metabolite accessibility	CRISPR activation, synthetic biology	[14,15]
Low cultivation efficiency	Reduced strain recovery	Advanced cultivation systems	[18,19,20]
Rediscovery of known metabolites	Reduced novelty	Metabolomics-guided dereplication	[16]
Low metabolite yield	Limited scalability	Chassis engineering and pathway optimization	[14,15]
Annotation overinterpretation	Misleading biosynthetic prediction	Functional validation and enzymology	[12,17]
Industrial scalability limitations	Delayed pharmaceutical translation	Biofoundries and systems biology	[12,17]
Poor pharmacokinetic properties	Limited clinical applicability	Medicinal chemistry optimization	[1,11]
Insufficient ecological interpretation	Incomplete functional understanding	Spatial metabolomics and synthetic ecology	[18,19,20]

## Data Availability

No new data were created or analyzed in this study. Data sharing is not applicable to this article.

## Abbreviations

The following abbreviations are used in this manuscript:

antiSMASH	antibiotics & Secondary Metabolite Analysis Shell
BAGEL	BActeriocin GEnome mining tooL
BGC	Biosynthetic Gene Cluster
CRISPR	Clustered Regularly Interspaced Short Palindromic Repeats
GNPS	Global Natural Products Social Molecular Networking
HPLC	High-Performance Liquid Chromatography
LC-MS/MS	Liquid Chromatography–Tandem Mass Spectrometry
MEP	Methylerythritol Phosphate pathway
MS/MS	Tandem Mass Spectrometry
NMR	Nuclear Magnetic Resonance
NRP(S)	Non-Ribosomal Peptide (Synthetase)
NRPS	Non-Ribosomal Peptide Synthetase
OSMAC	One Strain Many Compounds
PKS	Polyketide Synthase
PRISM	Prediction Informatics for Secondary Metabolomes
RiPPs	Ribosomally synthesized and Post-translationally modified Peptides
UHPLC-QTOF	Ultra-High Performance Liquid Chromatography–Quadrupole Time-of-Flight
WGS	Whole-Genome Sequencing

## References

[1] Fenical W., Jensen P.R. (2006). Developing a new resource for drug discovery: Marine actinomycete bacteria. Nat. Chem. Biol..

[2] Carroll A.R., Copp B.R., Davis R.A., Keyzers R.A., Prinsep M.R. (2019). Marine natural products. Nat. Prod. Rep..

[3] Barka E.A., Vatsa P., Sanchez L., Gaveau-Vaillant N., Jacquard C., Klenk H.-P., Clément C., Ouhdouch Y., van Wezel G.P. (2016). Taxonomy, physiology, and natural products of Actinobacteria. Microbiol. Mol. Biol. Rev..

[4] Bentley S.D., Chater K.F., Cerdeño-Tárraga A.M., Challis G.L., Thomson N.R., James K.D., Harris D.E., Quail M.A., Kieser H., Harper D. (2002). Complete genome sequence of the model actinomycete *Streptomyces coelicolor* A3(2). Nature.

[5] Demain A.L., Sanchez S. (2009). Microbial drug discovery: 80 years of progress. J. Antibiot..

[6] Berdy J. (2012). Thoughts and facts about antibiotics: Where we are now and where we are heading. J. Antibiot..

[7] Jensen P.R., Dwight R., Fenical W. (1991). Distribution of actinomycetes in near-shore tropical marine sediments. Appl. Environ. Microbiol..

[8] Zhou Q., Hotta K., Deng Y., Yuan R., Quan S., Chen X. (2021). Advances in Biosynthesis of Natural Products from Marine Microorganisms. Microorganisms.

[9] Bull A.T., Stach J.E. (2007). Marine actinobacteria: New opportunities for natural product search and discovery. Trends Microbiol..

[10] Rateb M.E., Ebel R. (2011). Secondary metabolites of fungi from marine habitats. Nat. Prod. Rep..

[11] Hughes C.C., Fenical W. (2010). Antibacterials from the sea. Chem. Eur. J..

[12] Kautsar S.A., van der Hooft J.J.J., de Ridder D., Medema M.H. (2021). BiG-SLiCE: A highly scalable tool maps the diversity of 1.2 million biosynthetic gene clusters. GigaScience.

[13] Blin K., Shaw S., Steinke K., Villebro R., Ziemert N., Lee S.Y., Medema M.H., Weber T. (2019). AntiSMASH 5.0: Updates to the secondary metabolite genome mining pipeline. Nucleic Acids Res..

[14] Rutledge P.J., Challis G.L. (2015). Discovery of microbial natural products by activation of silent biosynthetic gene clusters. Nat. Rev. Microbiol..

[15] Foldi J., Connolly J.A., Takano E., Breitling R. (2024). Synthetic Biology of Natural Products Engineering: Recent Advances Across the Discover–Design–Build–Test–Learn Cycle. ACS Synth. Biol..

[16] Wang M., Carver J.J., Phelan V.V., Sanchez L.M., Garg N., Peng Y., Nguyen D.D., Watrous J., Kapono C.A., Luzzatto-Knaan T. (2016). Sharing and community curation of mass spectrometry data with GNPS Natural Products Social Molecular Networking. Nat. Biotechnol..

[17] Kautsar S.A., Blin K., Shaw S., Weber T., Medema M.H. (2023). BiG-SLiCE 2 and scaling biosynthetic diversity analysis. Nat. Chem. Biol..

[18] Skropeta D. (2008). Deep-sea natural products. Nat. Prod. Rep..

[19] Jensen P.R. (2016). Natural products and the gene cluster revolution. Trends Microbiol..

[20] Sugimoto Y., Camacho F.R., Wang S., Chankhamjon P., Odabas A., Biswas A., Jeffrey P.D., Donia M.S. (2019). A metagenomic strategy for harnessing the chemical repertoire of the human microbiome. Science.

[21] Cane D.E., Walsh C.T., Khosla C. (1998). Harnessing the biosynthetic code: Combinations, permutations, and mutations. Science.

[22] Hertweck C. (2009). The biosynthetic logic of polyketide diversity. Angew. Chem. Int. Ed. Engl..

[23] Xu X., Huang X., Xu W. (2024). Marine actinomycetes-derived angucyclines and angucyclinones with biosynthesis and activity--past 10 years (2014–2023). Eur. J. Med. Chem..

[24] Liu Z., Sun W., Hu Z., Wang W., Zhang H. (2024). Marine *Streptomyces*-derived novel alkaloids Discovered in the Past Decade. Mar. Drugs.

[25] Kwon H.C., Kauffman C.A., Jensen P.R., Fenical W. (2006). Marinomycins A–D, Antitumor-Antibiotics of a New Structure Class from a Marine Actinomycete of the Recently Discovered Genus “Marinispora”. J. Am. Chem. Soc..

[26] Hughes C.C., Prieto-Davo A., Jensen P.R., Fenical W. (2008). The marinopyrroles, antibiotics of an unprecedented structure class from a marine *Streptomyces* sp.. Org. Lett..

[27] Olano C., Méndez C., Salas J.A. (2009). Antitumor compounds from marine actinomycetes. Mar. Drugs.

[28] Gribble G.W. (1998). Naturally occurring organohalogen compounds. Acc. Chem. Res..

[29] Winter J.M., Moore B.S. (2009). Exploring the Chemistry and Biology of Vanadium-dependent haloperoxidases. J. Biol. Chem..

[30] Wagner C., El Omari M., König G.M. (2009). Biohalogenation: Nature’s Way to Synthesize Halogenated Metabolites. J. Nat. Prod..

[31] Neumann C.S., Fujimori D.G., Walsh C.T. (2008). Halogenation strategies in natural product biosynthesis. Chem. Biol..

[32] Shigeno S., Kadowaki M., Nagai K., Hosoda K., Terahara T., Nishimura T., Hasegawa N., Tomoda H., Ohshiro T. (2024). New polycyclic tetramate macrolactams with antimycobacterial activity produced by marine-derived *Streptomyces* sp.. J. Antibiot..

[33] Shiomi K., Iinuma H., Hamada M., Naganawa H., Manabe M., Matsuki C., Takeuchi T., Umezawa H. (1986). Novel antibiotics napyradiomycins. Production, isolation, physico-chemical properties and biological activity. J. Antibiot..

[34] Thibodeaux C.J., Melançon C.E., Liu H.W. (2007). Unusual Sugar Biosynthesis and Natural Product Glycodiversification. Nature.

[35] Weymouth-Wilson A.C. (1997). The role of carbohydrates in biologically active natural products. Nat. Prod. Rep..

[36] Salas J.A., Méndez C. (2007). Engineering the glycosylation of natural products in actinomycetes. Trends Microbiol..

[37] Bernhardt R. (2006). Cytochrome P450 as versatile biocatalysts. J. Biotechnol..

[38] Podust L.M., Sherman D.H. (2012). Diversity of P450 enzymes in the biosynthesis of natural products. Nat. Prod. Rep..

[39] Kim H.J., Ruszczycky M.W., Choi S.H., Liu Y.N., Liu H.W. (2011). Enzyme-catalysed [4+2] cycloaddition is a key step in the biosynthesis of spinosyn A. Nature.

[40] Soohoo A.M., Cogan D.P., Brodsky K.L., Khosla C. (2024). Structure and Mechanisms of Assembly-Line Polyketide Synthases. Annu. Rev. Biochem..

[41] Grininger M. (2023). Enzymology of assembly line synthesis by modular polyketide synthases. Nat. Chem. Biol..

[42] Zhang Y., Li X., Wang J., Chen Q., Zhao P. (2024). Marine *Streptomyces* sp. PGC 39: A treasure trove of new antimicrobial agents, macrolidycin, and pyrachlomycin. Food Biosci..

[43] Ji C.H., Je H.W., Kim H., Kang H.S. (2024). Promoter engineering of natural product biosynthetic gene clusters in actinomycetes: Concepts and applications. Nat. Prod. Rep..

[44] Wang M., Chen L., Zhang Z., Wang Q. (2025). Recent advances in genome mining and synthetic biology for discovery and biosynthesis of natural products. Crit. Rev. Biotechnol..

[45] Asamizu S. (2025). Co-cultivation strategies for natural product discovery from actinomycetes: Unlocking silent secondary metabolism with mycolic acid-containing bacteria. World J. Microbiol. Biotechnol..

[46] Basnet B., Zhou Z.Y., Basnet R., Wei B., Wang H. (2026). Advances in natural product discovery: Strategies, technologies, and insights. Nat. Prod. Bioprospect..

[47] Wang Z., Yu J., Wang C., Hua Y., Wang H., Chen J. (2025). The Deep Mining Era: Genomic, Metabolomic, and Integrative Approaches to Microbial Natural Products from 2018 to 2024. Mar. Drugs.

[48] Seshadri K., Abad A.N.D., Nagasawa K.K., Yost K.M., Johnson C.W., Dror M.J., Tang Y. (2025). Synthetic Biology in Natural Product Biosynthesis. Chem. Rev..

[49] Li Y.J., Qiu M.H., Peng X.R. (2026). Revolutionizing microbial treasure troves: Innovative strategies for natural products discovery. Nat. Prod. Bioprospect..

[50] Simpson T.J. (2025). Synthetic biology routes to new and extinct natural products. RSC Chem. Biol..

[51] Heard S.C., Winter J.M. (2024). Structural, biochemical and bioinformatic analyses of nonribosomal peptide synthetase adenylation domains. Nat. Prod. Rep..

[52] Vasudevan D., Rao S.P., Noble C.G. (2013). Structural basis of mycobacterial inhibition by cyclomarin A. J. Biol. Chem..

[53] Kiefer A., Bader C.D., Held J., Esser A., Rybniker J., Empting M., Müller R., Kazmaier U. (2019). Synthesis of new cyclomarin derivatives and their biological evaluation towards *Mycobacterium tuberculosis* and *Plasmodium falciparum*. Chem. Eur. J..

[54] Kiefer A., Kazmaier U. (2019). Synthesis of modified β-methoxyphenylalanines via diazonium chemistry and their incorporation in desoxycyclomarin analogues. Org. Biomol. Chem..

[55] Fenical W., Jensen P.R., Palladino M.A., Lam K.S., Lloyd G.K., Potts B.C. (2009). Discovery and development of the anticancer agent salinosporamide A (NPI-0052). Bioorganic Med. Chem..

[56] Potts B.C., Albitar M.X., Anderson K.C., Baritaki S., Berkers C., Bonavida B., Chandra J., Chauhan D., Cusack J.C., Fenical W. (2011). Marizomib, a proteasome inhibitor for all seasons: Preclinical profile and a framework for clinical trials. Curr. Cancer Drug Targets.

[57] Macherla V.R., Mitchell S.S., Manam R.R., Reed K.A., Chao T.H., Nicholson B., Deyanat-Yazdi G., Mai B., Jensen P.R., Fenical W.F. (2005). Structure-activity relationship studies of salinosporamide A (NPI-0052), a novel marine derived proteasome inhibitor. J. Med. Chem..

[58] Potts B.C., Lam K.S. (2010). Generating a Generation of Proteasome Inhibitors: From Microbial Fermentation to Total Synthesis of Salinosporamide A (Marizomib) and Other Salinosporamides. Mar. Drugs.

